# Adaptive strategies of Egyptian crowfoot grass (*Dactyloctenium aegyptium* (L.) Willd): Germination responses to environmental factors

**DOI:** 10.1371/journal.pone.0345587

**Published:** 2026-05-07

**Authors:** Ahmad Zare, Elham Elahifard, Zahra Asadinejad

**Affiliations:** 1 Department of Plant Production and Genetics, Faculty of Agriculture, Agricultural Sciences and Natural Resources University of Khuzestan, Bavi, Mollasani, Iran; 2 M.Sc. Graduated in Weed Science, Department Plant Production and Genetics, Faculty of Agriculture, Agricultural Sciences and Natural Resources University of Khuzestan, Bavi, Mollasani, Khuzestan, Iran; Canakkale Onsekiz Mart University, TÜRKIYE

## Abstract

Egyptian crowfoot grass (Dactyloctenium aegyptium (L.) Willd.) is a troublesome C_4_ grass weed that competes with crops such as rice (Direct Seeded Rice), sugarcane and urban green space. The effects of constant and alternating temperatures, storage times, light, salinity, and water stress on germination of Egyptian crowfoot grass studied under laboratory conditions. Germination was stimulated by light, and seed germination percentage was higher under alternative temperatures compared to constant temperatures. Results also showed that germination percentage increased as the storage period lengthened. The highest germination percentage (97.33%) was observed under 30/20°C alternating temperature with light in seeds stored for 12 months. The time required to reach maximum germination was shorter under both constant and alternating temperatures for seeds stored for 12 months compared to those stored for 5 months and 2 months, respectively. Osmotic stress had moderate negative effects on germination, with 45% of the Egyptian crowfoot grass seeds able to germinate at −0.6 MPa,. Complete germination inhibition was observed at an osmotic potential of −1 MPa. The osmotic potential required for 50% inhibition of maximum germination was 0.49 MPa. Also results of salinity indicated that 23% of seeds germinated at sodium chloride (NaCl) concentration of 200 mM. A 50% reduction in germination of Egyptian crowfoot grass was caused 162 mM. To reduce the soil seed bank of this photoblastic weed, we recommend implementing deep tillage, applying straw mulch, and utilizing the false and stale seedbed techniques.

## 1. Introduction

Egyptian crowfoot grass (*Dactyloctenium aegyptium* (L.) Willd.) is a tufted, slightly stoloniferous annual or short-lived perennial grass that grows up to 75 cm in height [1]. It has been introduced as a C4 weed in 19 crops and 45 countries, [2] In different crops such as rice (*Oryza* *sativa* L*.*), cotton (*Gossypium hirsutum* L.), sugarcane (*Saccharum officinarum* L.), peanuts (*Arachis hypogaea* L.), corn (*Zea mays* L.), and vegetables, it is known to be a tenacious, and troublesome weed [3]. This species also grows in sunny or lightly shaded places in gardens, waste places, dikes, and embankments [4] The seed production ability is estimated at up to 66,000 seeds per plant, and propagates mainly due to the large production of seeds [3]. It is considered a weed in rice, sugarcane production regions and landscapes of southwestern Iran.

Germination and emergence of weed seeds are critical developmental stages that determine a weed’s success in agro-ecosystems [5]. The timing of seedling emergence, determined by germination-triggering abiotic cues, shapes the initial environmental conditions they experience and critically influences plant survival and establishment [6]. These processes are regulated by environmental factors, including temperature, soil pH, salinity, water availability, and seed burial depth [7]. Previous research indicates that these factors can either initiate or inhibit seed germination. Notably, temperature is a primary determinant of germination when other factors are non-limiting, with its effects varying among species within the same genus [8]. Temperature critically regulates seed germination rates through its role in overcoming seed dormancy. Determining optimal germination temperatures facilitates the prediction of peak emergence events within a season, informing targeted weed management strategies [7].

Studies have demonstrated that light and alternating temperature regimes serve as two critical environmental factors stimulating seed germination [9,10]. The Photochrome absorption levels in a seed can be used to evaluate the competitive pressure in its immediate environment and early detection of competition before germination improves seedling survival rates [11]. In an environment with minimal competition, the seed senses a higher ratio of red light compared to infrared light, encouraging germination, Seeds sprouting in complete darkness may have elevated levels of far-red phytochromes in their embryonic tissue, which assist in sensing intense competition [12]. By determining that a weed is positively photoblastic, light-limiting management techniques, such as mulching with crop residues, can be applied [13].

Water stress can delay, reduce, or inhibit weed seed germination. However, the ability of certain weed species to germinate under low-moisture conditions provides a competitive advantage, enabling them to outperform many crops in germination and growth [7]. Salinity is a major environmental stressor that reduces germination rates, delays germination onset, and impairs seedling establishment, ultimately limiting agricultural productivity and sustainability in arid and semi-arid regions [14]. After-ripening refers to physiological and biochemical changes occurring in dry (non-imbibed) seeds following dispersal, which break seed dormancy and enhance germination capacity and After-ripening is significantly influenced by storage conditions and their duration [15]. The process may be attributed to Changes in seed chemical composition, decreased abscisic acid (ABA) levels during storage and increased Gibberellic acid concentrations [16]

Plentiful seed output is a hallmark of thriving weeds. A robust soil seedbank gives weeds an advantage over crops and native plants, particularly when they grow faster than desired species. These substantial seed stores result in ongoing control challenges [17]. A thorough comprehension of the biological mechanisms behind seed germination, particularly under varying environmental conditions, is fundamental for developing weed management strategies. The ability of weed seeds to germinate and successfully emerge from the soil plays a pivotal role in their persistence and colonization within agroecosystems [18–20]. Understanding the relationship between environmental factors and plant physiology is crucial for analyzing and predicting ecological dynamics. This interaction is shaped by local climatic conditions, including latitude, elevation, soil moisture, temperature and precipitation patterns, light availability, and photoperiod (day length) [21]. Understanding seed dormancy, germination and its relationship with environmental factors is essential for predicting germination timing and implementing effective weed management strategies [22].

Weed seed populations exhibit significant divergence in germination behavior across heterogeneous geographic origins and habitat types [23,24]. Germination responses of Egyptian crowfoot grass populations exhibit significant geographic variation. While US populations germinate across 15–40°C peaking at 94% under 35/25 C alternating day/night temperatures [25], Philippine populations show ≤20% germination in darkness at 30/20C night [2]. African populations achieve 100% germination in both light and dark regimes [26]. In response to water stress, Egyptian crowfoot grass seeds could tolerate water stress up to −0.6 MPa, [2,25]. [27] reported that the Egyptian crowfoot grass population could survive with a low growth rate at salinities of up to 50% artificial seawater. Weed seeds may also have different germination requirements if they mature under different environmental conditions.

A key methodological distinction concerns post-harvest storage duration: 6 months for the US population versus 9 months for the Philippine population, aligned with respective maternal plant rainfall regimes prior to testing. Storage intervals (post-ripening) may significantly influence germination capacity and velocity, potentially altering optimal weed management timing [28].

The agricultural landscape of Khuzestan Province has undergone significant transformation due to climate change impacts, particularly increasing thermal regimes and diminishing hydrological resources. These environmental shifts have necessitated the adoption of direct-seeded rice methodologies as an alternative to traditional paddy-based cultivation. Consequently, there emerges a critical research imperative to elucidate the germination dynamics of autochthonous populations of Egyptian crowfoot grass, a competitive weed species exhibiting increasing proliferation in Iranian rice production systems.

This study expands on prior regional observations by quantifying germination plasticity in Iranian populations under projected climate scenarios. The objectives of this research were to determine (a) the effect of temperature and light in three storage times (2, 5 and 12 months after rainfall of mother plant) and (b) the effect of osmotic potential, and salinity stresses on seed germination of Egyptian crowfoot grass.

## 2. Results

### 2.1. Temperature, storage and light

A significant three-way interaction among temperature, storage, and light was detected, along with significant two-way interactions between temperature × storage and temperature × light on seed germination (Table 1).

**Table 1:** Analysis of variance for seed germination of Egyptian crowfoot grass (*Dactyloctenium aegyptium*) at constant temperature combined with storage times and light conditions.

Source of variation	df	MS	F-Value	P-Value
Temperature	7	8227.01	911.57	**
Storage	2	1792.3	198.59	**
Light	1	18741.40	2076.58	**
Temperature × storage	14	223.90	24.81	**
Temperature × light	7	1788.70	198.19	**
Light × storage	2	15.85	1.75	NS
Temperature × storage × light	14	18.10	2.01	**
Error	336	9.00		
C.V. (%)	21.75			

**indicate significance at the 0.01 probability level.

**Fig 1:**
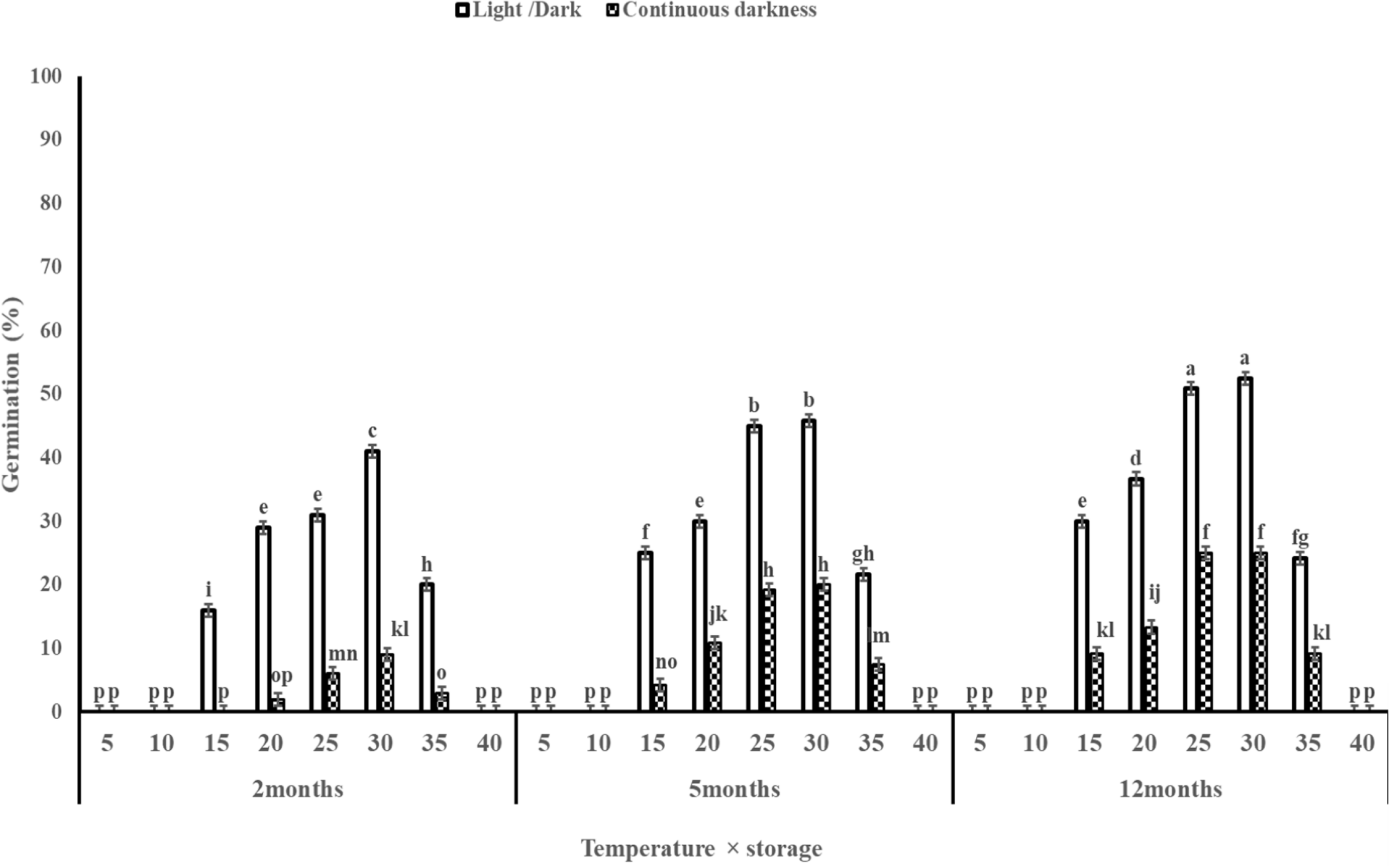
Effect of interaction constant temperatures, storage times and light regimes on seed germination of Egyptian crowfoot grass. Nails on the vertical bars represent the standard error of the means.

The highest germination percentage was observed at 30 °C across all storage times (Fig 1). Seeds stored for two months exhibited consistently lower germination than the longer storage times (five and 12 months) at all constant temperatures (Fig 1). Maximum germination (52.5%) occurred after 12 months of storage at 30 °C under light/dark conditions, whereas no germination was detected at 5, 10, and 40 °C. Notably, germination of seeds stored for two months at 25 °C under light/dark conditions (31%) did not differ significantly from that of seeds stored for 12 months at 15 °C (30%).

**Fig 2:**
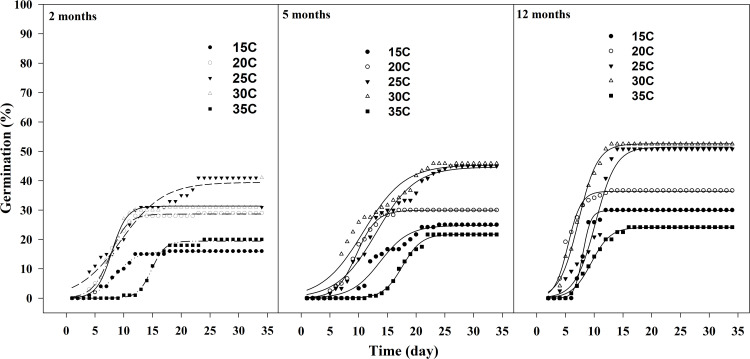
Effect of constant temperatures and storage times on cumulative germination of Egyptian crowfoot grass in light/dark regime.

Egyptian crowfoot grass germination was significantly influenced by temperature, storage and light (Fig 1). When exposed to light, seed germination was consistently higher than in darkness across all constant temperatures at 5 and 12 months storage than 2 months (Fig 1). Under light conditions, germination ranged from 16 to 52.5%, depending on temperature and storage times. In contrast under dark conditions germination was much lower, ranged from 2 to 25% that was the difference statistically significant (Fig 1). Based on the small seed size, light appears to be the predominant factor regulating germination in this weed species. Also Temperatures ranging from 25 to 30 °C can significantly enhance the germination of this weed. Given the critical role of temperature in regulating germination, it is evident that conditions within this thermal range are particularly conducive to germination success.

with increasing seed storage times in light/dark regime, the maximum germination percentage improved, and the time required to reach maximum germination was shorter compared to seeds stored for two months (Fig 2 and Table 2). For seeds stored two months of storage, estimating the confidence limits of the averages revealed that the maximum percentage of seed germination at different constant temperatures was significant differences from each other (P ≤ 0.05).

**Table 2:** Estimated parameters of the three-parameter sigmoidal equation for cumulative germination in storages times and constant temperatures.

Storage (month)	Temperature	G_max_ (%)	G_rate_	T_50_ (day)	R^2^
2	15	15.93 (±0.12^*^)	1.82 (±0.08)	8.63 (±0.18)	0.99
	20	28.64 (±0.14)	1.56 (±0.06)	7.56 (±0.07)	0.99
	25	39.44 (±0.90)	3.70 (±0.40)	9.55 (±0.44)	0.94
	30	31.35 (±0.40)	1.13 (±0.15)	7.91 (±0.17)	0.97
	35	19.44 (±0.17)	1.19 (±0.08)	14.84 (±0.09)	0.98
5	15	24.75 (±0.48)	2.51 (±0.25)	13.72 (±0.30)	0.97
	20	29.97 (±0.15)	1.75 (±0.06)	9.63 (±0.07)	0.99
	25	44.65 (±0.72)	3.39 (±0.23)	13.06 (±0.27)	0.98
	30	45.38 (±1.06)	3.58 (±0.38)	10.85 (±0.43)	0.95
	35	21.77 (±0.16)	1.66 (±0.07)	17.63 (±0.08)	0.99
12	15	29.84 (±0.17)	0.70 (±0.05)	8.00 (±0.05)	0.99
	20	36.41 (±0.26)	1.15 (±0.08)	5.45 (±0.09)	0.98
	25	51.42 (±0.67)	1.78 (±0.16)	10.23 (±0.19)	0.97
	30	52.58 (±0.27)	1.65 (±0.07)	7.27 (±0.08)	0.99
	35	24.12 (±0.13)	1.66 (±0.07)	9.49 (±0.08)	0.99

Abbreviation: G_max_ is the maximum germination (%), T_50_ is the times (day) required to reach 50% of the maximum germination, and G_rate_ indicates the slope.

*The numbers in parentheses are standard errors.

In comparison, seed germination exhibited a significant differences across constant temperatures for five months of storage times (P ≤ 0.05). However Seed germination at 25 and 30 ºC after 12 months of storage showed no significant different (P ≤ 0.05). notably the highest germination percentages were recorded for seeds stored for 12 months (Table 2).

As shown in Table 2, there was no significant difference in the T_50_ value between temperatures 15 and 25 ºC, or between 20 and 30 ºC after 2 months of storage times. Although, the T_50_ value at 35 ºC differed significantly from all other constant temperatures. For storage times of five and 12 months, the estimation of confidence limits for the T_50_ values revealed significant difference across all constant temperatures (P ≤ 0.05).

The duration of the lag phase at 30, 25, and 20 ºC was shorter than at 15 and 35 ºC, especially at 12 months of storage compared to 5and 2 months storages (Fig 2- sigmoid equation). At 35 ºC, the onset of germination occurred 12 days after seed imbibition for storage times of two and five months, whereas seed germination was observed within less than five days for seeds stored for 12 months. The time required to reach maximum germination for seeds stored for 12 months at 30 °C was less than 10 days, compared to more than 20 days for seeds stored for two and five months (Fig 2).

At 35 ºC, seeds stored for two and five months had the highest T_50_ values (14 and 17 days respectively). In contrast, the T_50_ it was nine days for seeds that were stored for 12 months at 35 ºC. At all storage treatments, a minimum of T_50_ was estimated for incubated seeds at 20 ºC (5.45–7.56 days) (Table 2). Also, the maximum cumulative germination percentage in the seeds stored for 12 months was higher than in other storage times (Fig 2 and Table 2).

### 2.2. Alternating temperatures, storage and light

No significant interaction effects were observed among the three experimental treatments or between temperature and storage conditions on germination. Interaction effects temperature × light and light × storage were significant on seed germination of Egyptian crowfoot grass (Table 3).

**Table 3:** Analysis of variance for seed germination of Egyptian crowfoot grass (*Dactyloctenium aegyptium*) at alternating temperatures combined with storage times and light conditions.

Source of variation	df	MS	F-Value	P-Value
Temperature	2	7691.3	259.58	**
Storage	2	1216.6	41.06	**
Light	1	68503.7	2312.00	**
Temperature × storage	4	20.1	0.68	NS
Temperature × light	2	701.0	23.66	**
Light × storage	2	142.8	4.82	**
Temperature × storage × light	4	36.1	1.22	NS
Error	90	29.6		
C.V. (%)	10.99			

**indicate significance at the 0.01 probability level.

**Fig 3:**
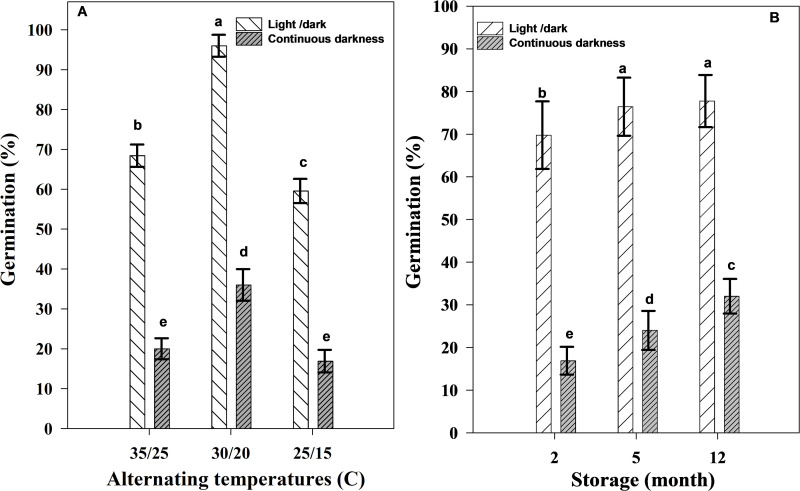
Effect of alternating temperatures (A) and storage times (B) under light and dark regimes on seed germination of Egyptian crowfoot grass. Nails on the vertical bars represent the standard error of the means.

As shown in Fig 3A, seed germination under continuous darkness was consistently reduced compared to the light/dark regime across all alternating temperatures. The highest seed germination (96%) was recorded at 30/20 °C under light conditions (Fig 3A). In the light/dark regime, seed germination was 68% at 35/25 °C and 59% at 25/15 °C. Notably, for seeds incubated in complete darkness, germination were significantly lower, with 36% at 30/20 °C, 20% at 35/25 °C, and 16% at 25/15 °C.

Germination under light/dark regime (69%, 76%, 77%) significantly exceeded (p < 0.01) that under continuous darkness (16%, 24%, 32%) across all storage periods (2, 5, 12 months) (Fig 3B). Twelve months of storage under continuous darkness resulted in a 100% increase in germination compared to the 2-month storage times (Fig 3B).

**Fig 4:**
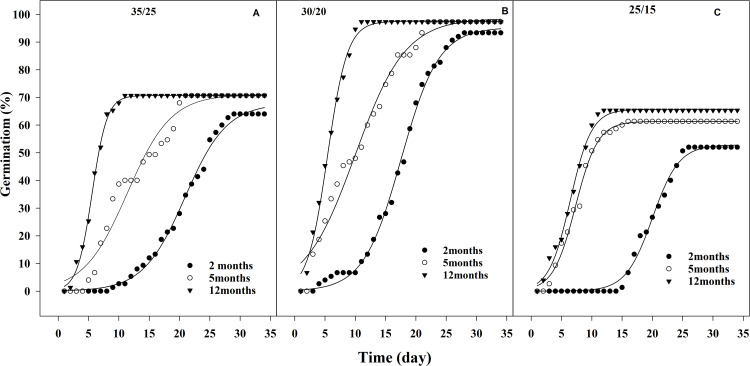
Effect of alternating temperatures (35/25 ºC(A), 30/20 ºC (B), and 25/15 ºC (C)) and storage times on cumulative germination of Egyptian crowfoot grass in light/dark regime.

Across all storage times, the cumulative seed germination at 30/20 exceeded 95%, while at 35/25 °C and 25/15 °C, the values ranged from 67% to 71% and 52% to 65%, respectively (Fig 4). Cumulative germination analysis revealed that the lag phase for seeds stored for two months was longer than for those stored for five and 12 months at all tested alternating temperatures (Fig 4).

According parameter sigmoidal equation in light/dark regime treatment, shown in Table 3, seed germination was highest (95.30–98.56%) at the alternating temperature of 30/20 °C. The difference in germination percentage at this temperature was statistically significant compared to other alternating temperatures, as indicated by the estimated confidence limits (P ≤ 0.05) (Table 4).

**Table 4:** Estimated parameters of three-parameter sigmoidal equation for cumulative germination in storage and alternating temperatures.

Temperature (ºC)	Storage (month)	G_max_	G_rate_	T_50_	R^2^
35/25	2	67.77 (±1.02^*^)	3.44 (±0.14)	20.90 (±0.18)	0.99
	5	71.06 (±1.45)	3.59 (±0.32)	11.41 (±0.37)	0.96
	12	70.69 (±0.20)	1.23 (±0.03)	5.58 (±0.04)	0.99
30/20	2	95.30 (±0.81)	3.10 (±0.40)	17.55 (±0.12)	0.99
	5	98.56 (±1.33)	4.15 (±024)	9.98 (±0.26)	0.98
	12	97.53 (±0.41)	1.64 (±0.06)	5.39 (±0.06)	0.99
25/15	2	52.85 (±0.68)	2.19 (±0.12)	20.00 (±0.14)	0.99
	5	61.32 (±035)	1.84 (±0.08)	7.31 (±0.09)	0.99
	12	65.69 (±0.30)	1.75 (±0.06)	6.46 (±0.07)	0.99

Abbreviation: G_max_ is the maximum germination (%), T_50_ is the times (day) required to reach 50% of the maximum germination, and G_rate_ indicates the slope.

*The numbers in parentheses are standard errors.

The time to achieve 50% maximum germination (T₅₀) ranged from 5 to 20 days for seeds stored 2–12 months under 30/20°C alternating temperatures (Table 4). T₅₀ values for 12-month-stored seeds were significantly reduced (15 days shorter; P < 0.05) compared to those stored for 2 or 5 months. At 2 months storage, T₅₀ reached 20.90, 17.55, and 20.00 days under 35/25°C, 30/20°C, and 25/15°C regimes, respectively. Corresponding values for 5month storage were 11.41, 9.98, and 7.31 days, decreasing further to 5.58, 5.39, and 6.46 days after 12 months storage (Table 4).

### 2.3. Salinity and osmotic potential

**Fig 5:**
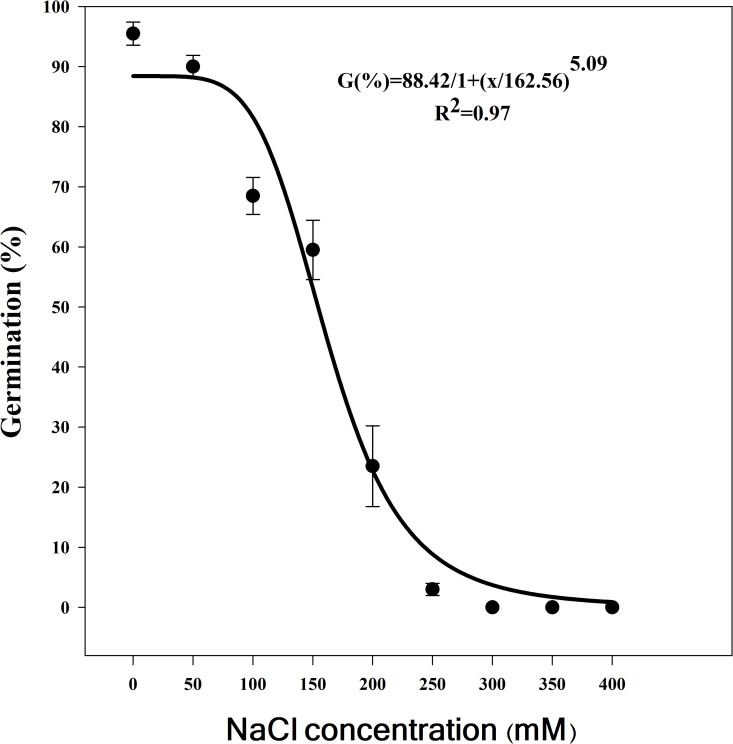
Effect of NaCl concentration (mM) on seed germination of Egyptian crowfoot grass. The bold line represents a three-parameter logistic model fitted to the data. Vertical bars represent the standard error of the mean.

Based on the fitted three-parameter logistic equation for seed germination at different NaCl concentrations, the highest seed germination (95%) was observed in the control treatment (0 mM NaCl) (Fig 5). Germination declined with increasing salinity, and at 300 mM NaCl, germination was completely inhibited. seed Germination decreased to 68% and 23% at NaCl concentrations of 100 mM and 200 mM, respectively, while at 50 mM NaCl, germination remained high at 90%. The NaCl concentration required to induce a 50% reduction in seed germination (X_50_) was estimated at 162 mM. Germination of Egyptian crowfoot grass decreased sharply as NaCl concentrations exceeded 150 mM, with notable seed germination of 23% and 3% at 200 and 250 mM NaCl, respectively.

**Fig 6:**
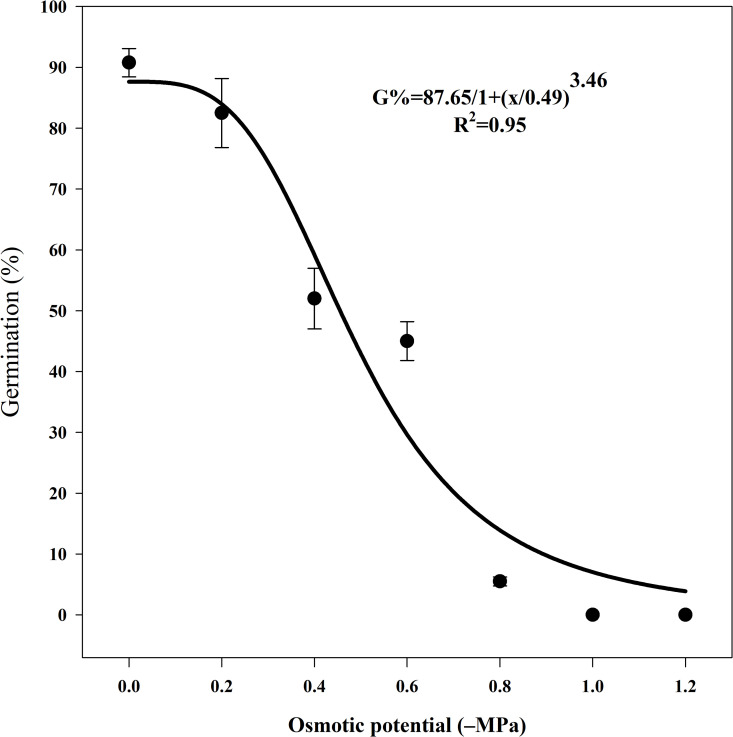
Effect of osmotic potential (-MPa) on seed germination of Egyptian crowfoot grass. The bold line represents a three-parameter logistic model fitted to the data. Vertical bars represent the standard error of the mean.

With increasing drought stress (i.e., a decrease in osmotic potential) from 0 to −0.6 MPa, seed germination decreased from 90% to 45% (Fig 6). At an osmotic potential of −0.8 MPa, only 5% of seeds germinated, while germination was completely inhibited at −1 MPa (Fig 6). The osmotic potential required for 50% inhibition of maximum germination was −0.49 MPa. A 30% reduction in germination was observed when the osmotic potential decreased from −0.2 to −0.4 MPa, while a 40% reduction occurred as the osmotic potential decreased from −0.6 to −0.8 MPa.

## 3. Discussion

### 3.1. Constant, alternative temperature, light and storage

The results of constant and alternative temperatures demonstrated, the seeds were exposed to light, germination was significantly higher than in darkness at all temperatures. Under the light/dark regime, there was a statistically significant difference between 30 ºC (46%) and other constant temperatures: 15 ºC (23%), 20 ºC (31%), and 35 ºC (21%). In the continuous darkness treatment, seed germination ranged from 4 to 18% depending on the temperatures (Fig 2b). Maximum germination occurred under light conditions at 30/20°C alternative temperatures. These results indicate that under Khuzestan’s climate, Egyptian crowfoot grass exhibits peak germination during May (32.3/22.7 C day/night)-June (38.3/31.1 C day/night) when average temperatures approximate this optimal regime. Conversely, July’s elevated temperatures (exceeding 35°C daytime) significantly suppress germination rates compared to June conditions. Storage times also revealed that longer periods led to higher germination percentages, and the time required to reach maximum germination, particularly under alternating and constant temperatures was shorter in the 12 months treatment compared to the 5 and 2 month treatments. This suggests that seeds rain filled from the mother plant in the previous year and remaining in the soil for about one year germinate more rapidly upon receiving the necessary temperature and light cues. Therefore, the timing of application in weed management programs may also be influenced.

The findings indicate that, alongside temperature- a well-documented driver of weed seed germination—light also plays a critical role, particularly in small-seeded weed species. The results of Constant and alternative temperatures revealed, the seed germination of Egyptian crowfoot grass is stimulated by light, indicating that the species is positively photoblastic. Gibberellin production, regulated by phytochrome, plays a crucial role in mediating plant responses to light. Exposure to red light converts phytochrome from its inactive Pr form which inhibits germination into the active Pfr form, promoting germination. In contrast, far-red light reverses this process, reverting Pfr back to the inhibitory Pr state [29].

High temperatures suppress seed germination in many plant species, primarily due to elevated levels of endogenous abscisic acid (ABA). This increase in ABA is mediated by the transcriptional activation of genes involved in ABA signaling pathways [30]. Also Under high-temperature conditions, seed sensitivity to abscisic acid (ABA) increases, partly because the embryo is unable to deactivate ABA and because dissolved oxygen levels decline in water-imbibed seeds [31].

The findings indicate the widespread prevalence of this weed species throughout Khuzestan from early to late spring, demonstrating significant invasive potential in direct-seeded rice (DSR) fields, sugarcane, and urban green spaces. The photoblastic nature of Egyptian crowfoot grass carries ecological significance, as light serves as a soil depth indicator that promotes higher germination rates in surface-positioned seeds compared to deeply buried ones [32].

Small-seeded species such as Egyptian crowfoot grass may possess insufficient seed reserves to support emergence from deeper soil layers. Other potential factors limiting germination of deeply buried seeds include light deprivation, hypoxia (oxygen deficiency), and reduced gaseous diffusion rates at greater depths [2,33]

The findings suggest that agricultural practices involving shallow burial of weed seeds may enhance seedling emergence. In no-tillage systems, where seeds remain predominantly on the soil surface, a greater density of emerged seedlings is observed. This early emergence promotes premature weed-crop competition, necessitating timely intervention in integrated weed management strategies [34]. Conversely, deep tillage reduces light penetration to weed seeds, thereby limiting germination and contributing to a decline in the soil seed bank. Nevertheless, a thorough understanding of the seed bank’s history is essential. Previous deep plowing may have sequestered substantial quantities of weed seeds, which could become re-exposed through subsequent soil disturbance. Mitigating this risk requires insights into the persistence and viability of weed seeds within the soil matrix [35].

An additional validated weed management strategy employs plant-derived mulches within integrated cropping systems. In Khuzestan Province’s predominant wheat-rice rotation system, conservation tillage with wheat residue retention provides multifunctional suppression through: 1-Physical suppression of seedling emergence by acting as a surface barrier; 2-Reduction in photosynthetic photon flux density (PPFD), which constrains phytochrome-regulated germination in photoblastic weed species; and 3- Gradual release of allelochemicals, which interfere with root development and cellular processes in emerging weed seedlings [36–38].

False or stale seedbed techniques represent ancillary strategies within integrated weed management frameworks [39]. This approach entails precisely timed tillage during periods when diurnal temperatures range 30/20 °C (day/night), facilitating the upward redistribution of weed seeds to the soil surface. Subsequent irrigation and light exposure induce germination particularly those seeds that have persisted in the soil seed bank for prolonged durations, allowing for the preemptive elimination of weed seedlings through mechanical means (e.g., disking, flaming) or chemical control using non-selective herbicides such as paraquat or glyphosate. By targeting early-stage weeds before crop establishment, the false or stale seedbed techniques substantially depletes the soil seed bank and supports long-term weed suppression [40].

The results of the effect of constant temperatures on seed germination of Egyptian crowfoot grass (collected from the United States) indicate the maximum germination occurred at 30 ºC (42%). additionally, seed germination at 35, and 15 ºC, was lower than 20, 25, and 30 ºC temperatures, when seeds were stored for six months at 28 ºC [25].

Additionally, after ripening due to storage has also had a positive effect on seed germination. For instance, the American and Philippine populations exhibited maximum seed germination at alternating day/night temperatures of 25/15 °C (92%) and 30/20 °C (94%), respectively [2,25]. In contrast, the Iranian population showed the highest seed germination (97.33%) at 30/20 °C after 12 months of storage, while the lowest rate (52.85%) was observed at 25/15 °C after two months of storage times. Germination of freshly harvested seeds was stimulated by both light and storage, suggesting that after-ripening is a prerequisite for germination in this species. However, the response to after-ripening varies significantly among species. For example, seeds of *Echinochloa colona* (L*.*) are stimulated by light but are not influenced by storage times [41]. Seed germination after 5 and 12 months of storage was higher than after 2 months under both light and dark regimes (Fig 3b). Specifically, seeds stored for 12 months exhibited the highest germination (77%) under light conditions, compared to those stored for 5 and 2 months (76% and 69%, respectively) (Fig 3b). Increasing the storage times from 2 to 12 months enhanced seed germination from 16% to 32% in darkness, while germination under the light/dark regime ranged from 69% to 77% (Fig 3b).

Similarly, seeds of Egyptian crowfoot grass from the United States, stored at room temperature (28 °C) for 6 months, exhibited higher seed germination (94.7%, 86.6%, and 86.3%) when exposed to alternating temperatures of 35/20, 30/20, and 30/15 °C, compared to 25/10 °C (67.8%) [25]. The time required to reach maximum germination was less than 3 days at higher alternating temperatures. In contrast, seed germination in a population collected from the Philippines was higher at 25/15 °C (92%) than at 30/20 °C (70%) and 35/25 °C (44%). It can be concluded that seed germination significantly decreased (17–31%) under dark conditions across the three alternating temperatures [2]. Chauhan and Johnson (2009a) suggested that goosegrass seeds lose sensitivity to light after three months of after-ripening. The germination responses of Egyptian crowfoot grass are similar to those of other summer grass weed species, such as liver seedgrass (*Urochloa panicoides* P. Beauv.) and junglerice (*E. colona*), whose seeds germinate at alternating temperatures ranging from 25/15 °C to 35/25 °C [41–43].

Research on eight *Euphorbia* species demonstrated that seed germination percentage increased with longer storage times. Seeds stored for more than 150 days showed significantly higher germination rates compared to those stored for fewer than 150 days. Additionally, the study found that freshly harvested seeds of *Eriochloa villosa* (Thunb.) exhibited lower germination potential, germination rate and germination index than stored seeds [44].

The effects of dry storage on *Isatis violascens* seed germination revealed distinct temporal patterns. At storage initiation (time zero), seed germination were minimal, with only 1% of silicles and 3% of isolated seeds viable. After six months of dry storage under optimal conditions (darkness, 2–5°C), germination rates increased to 11% for silicles and 24% for isolated seeds. Continued after-ripening between 6–12 months significantly enhanced germination capacity, reaching 38% for silicle-contained seeds and 71% for isolated seeds by the end of the 12-month storage times [45].

The research of seven temperatures (10–40°C at 5°C intervals) and seven after-harvest periods (30–540 days after harvest) revealed distinct temperature-dependent germination responses among nine *Amaranthus* species, with most exhibiting optimal germination (70–100%) at 35°C, while negligible germination occurred at 10–15°C for *A. albus*, *Amaranthus*. *deflexus, Amaranthus*. *graecizans*, *and Amaranthus*. *lividus*. Notably, *Amaranthus*. *cruentus, Amaranthus*. *hybridus*, and *Amaranthus*. *retroflexus* showed intermediate tolerance, germinating at 15°C (>60%) In contrast, *Amaranthus*. *blitoides* and *Amaranthus*. *viridis* uniquely required alternating temperatures (30/10 °C) and light exposure to achieve high germination (>90%), underscoring their adaptation to fluctuating environmental conditions. After-ripening duration significantly influenced germination in *A*. *cruentus*, *A*. *hybridus*, and A. *retroflexus*, but had marginal effects on *A*. *graecizans and A*. *lividus*, highlighting species-specific dormancy strategies [46].

### 3.2. Salinity and osmotic potential

Germination percentages declined with increasing salinity: 90% at 50 mM, 68% at 100 mM, 59% at 150 mM, and 23% at 200 mM. Based on the logistic equation, the X₅₀ (salinity reducing maximum germination by 50%) was estimated at 162 mM. Germination of Egyptian crowfoot grass decreased sharply at NaCl concentrations exceeding 150 mM, indicating that this species is more tolerance to salinity than other grasses, such as *E. colona* (106 mM), *Eleusine indica* (L.) Gaertn. (74 mM) and *Leptochloa chinensis* (L.) Nees (50 mM) [19].

High salinity reduces the osmotic potential of water in the soil, ultimately leading to decreased water absorption by dry seeds (imbibition process). Additionally, excessive uptake of sodium (Na⁺) and chloride (Cl⁻) ions from the soil causes ionic stress and toxicity, thereby disrupting biochemical processes such as nucleic acid and protein metabolism, energy production, and respiration [47]. Salinity disrupts the balance of key nutrients and hormones particularly gibberellin (GA) and abscisic acid (ABA) during seed germination. High salinity levels can delay germination or, depending on the plant’s salt tolerance, completely inhibit it. Under severe salt stress, the delicate balance between reactive oxygen species (ROS) production and scavenging is disturbed, leading to the accumulation of harmful ROS such as hydroxyl radicals, superoxide, and hydrogen peroxide. These ROS oxidatively damage critical macromolecules (e.g., proteins, carbohydrates, nucleic acids, and lipids) and disrupt cellular structures (e.g., membranes), ultimately impairing or preventing germination [48].

Limited information is available on the response of this weed to salt stress. Egyptian crowfoot grass seeds exhibited varying germination behaviors across different populations. For instance, when exposed to seawater concentrations, the Otta population showed a gradual decline in germination, with the lowest seed germination observed at 100% seawater concentration. In contrast, the Lagos population exhibited a sharp decline in germination, with complete inhibition at 20% seawater concentration [26]. Similarly, under salinity conditions, less than 20% of *Brachiaria eruciformis* seeds germinated at 150 mM NaCl [49]. Japanese brome (*Bromus japonicus*) demonstrated higher salinity tolerance, with an X_50_ value predicted at 202 mM NaCl [50]. According to Holm et al. (1977), the ability of this plant to produce seeds suggests that its germination capacity could significantly contribute to the establishment of dynamic populations in saline environments [3].

The osmotic potential required for 50% inhibition of maximum germination was −0.49 MPa, although some seeds germinated at −0.8 MPa. However, no germination occurred when the osmotic potential was greater than −0.8 MPa. In comparison, the osmotic potential for 50% inhibition of maximum germination in the Philippine population was −0.23 MPa [2]. Notably, no germination occurred at −0.8 ≤ MPa for American and Philippine populations [2,25]. An osmotic potential greater than −0.8 MPa completely inhibits the germination of both grass and broadleaf weeds [24], while osmotic potential ranging from −0.09 to −0.32 MPa reduces weed germination by 50% compared with the unstressed condition [51] The ability of the Iranian population to germinate at −0.8 MPa suggests greater drought tolerance compared to the American and Philippine populations.

Soil moisture influences both the timing of weed emergence and the number of weed seedlings emerging. In dry conditions, insufficient water can be the primary constraint on seed germination [52]. Water potential is a primary driver of water uptake, including seed imbibition; drought stress reduces water potential, thereby limiting uptake [53].

These findings suggest that seeds of certain species remain dormant until sufficient moisture becomes available, a mechanism that accounts for the mass emergence of seedlings at the onset of the rainy or monsoon season, which typically aligns with the start of the cropping period. This behavior likely reflects an adaptive strategy to delay germination until favorable conditions arise, thereby enhancing seed longevity within the seed bank [19].

Finally, this ability and tolerance to salinity and osmotic potential allows this weed to have a distinct competitive edge over crops like rice (direct seeded), sugarcane and urban green space, which may be subjected to stress in such environments.

## 4. Conclusion

In summary, the present study demonstrated that this weed exhibits distinct germination responses to constant temperatures, alternating temperatures, light, and storage times. Maximum germination (97%) was achieved under light conditions at 30/20°C alternating temperatures after 12 months of storage, confirming its photoblastic nature. Increased storage times (after-ripening) following rainfall from the mother plant significantly reduced the time required to reach peak germination, indicating potential for rapid early-season establishment. Based on the X₅₀ parameter, which quantifies the salinity and drought thresholds required to suppress germination by 50%, this weed exhibits considerable tolerance to both stress conditions. Germination persists even at salinity 150 mM (60%) and an osmotic potential of −6 bars (45%). Given climate change-induced shifts and effect on salinity, and drought stress in Khuzestan Province, expansion of this weed in summer crops is highly probable. Consequently, integrated management strategies including deep tillage, wheat straw mulching, and false or stale seedbed techniques should be implemented to deplete its soil seed bank.

## 5. Materials and methods

### 5.1. Seed collection and storage

Seeds of Egyptian crowfoot grass were obtained from Agricultural Sciences and Natural Resources University of Khuzestan located in the Mollasani district of Bavi County, Iran (31°35’56.1"N 48°53’18.8"E) during autumn 2017. Mature seeds were collected from a minimum of 100 individual plants to ensure genetic diversity and representativeness. After collection spikes were air-dried at 25 °C under ambient conditions for one week, then threshed and sieved to separate the grains seeds were cleaned, and placed in a paper bag for storage, allowing them to dry until reaching 11% moisture content and finally stored at room temperature (25 ºC) for two, five, and twelve months before their use in three experiments. No dormancy-breaking treatment was applied; seeds were stored at room temperature for durations specified by the experimental design.

### 5.2. Effect of constant temperatures, storage and light on germination

Eight constant temperatures (5, 10, 15, 20, 25, 30, 35 and 40 ºC) under two light regimes (light/dark (16-h photoperiod/8-h dark) and continuous darkness) and 3 storage times (2, 5 and 12 months after rainfall of mother plants) were tested. A light intensity of 185 μmol m ⁻ ² s ⁻ ¹ was maintained in the germination chambers using fluorescent lamps.

Petri dishes with a diameter of 10 cm, lined with two layers of Whatman filter paper, were used. Thirty seeds were placed in each Petri dish. At the beginning of the experiment, 5 mL of distilled water was added to Petri dishes, and seed germination was counted daily until 34 days with visible protrusion of the radicle to (2 ≥ mm) used as the criterion germination [54]. To create darkness, Petri dishes were wrapping in two layers of aluminum foil. To maintain optimal moisture under dark conditions, seeds were fully shielded from white light. Petri dishes were inspected solely under green safe light to preserve complete darkness during observations.

### 5.3. Effect of alternating temperatures, storage and light on seed germination

Three alternating temperatures 35/25, 30/20, and 25/15 ºC under two light regimes light/dark and continuous darkness) and 3 storage times (2, 5 and 12 months after rainfall of mother plants) were selected (The selected temperature regimes reflect the thermal variations observed in Khuzestan). The experimental procedure was similar to that used for constant temperatures.

### 5.4. Effect of osmotic potential and salinity on seed germination

Seven concentrations of polyethylene glycol 6000 (0, −0.2, −0.4, −0.6, −0.8, −1, and 1.2 MPa) were assessed to determine the effect of osmotic potential on seed germination. The solution was prepared by dissolving polyethylene glycol 6000 in distilled water based on 25 C temperature [55].

Also, nine concentrations of sodium chloride (NaCl) (0, 50, 100, 150, 200, 250, 300, 350, and 400 mM) were selected to determine the response of seed germination to salt stress. Thirty seeds were placed in each Petri dish, and 5 mL of the respective solutions were added to each dish. Petri dishes covered with a plastic bag to prevent water loss. Petri dishes were placed at 30/20 ºC (optimal temperature based on alternating temperatures) under the light/dark regime. germination was counted daily for 21 days.

### 5.5. Statistical analyses

A completely randomized design (CRD) was used for all experiments, with four replications per treatment. Prior to conducting ANOVA using SAS software (version 9.4), we verified the assumptions of normality (Shapiro-Wilk test) and homogeneity of variance for each experiment. No data transformation was required as all assumptions were satisfied. A three-way ANOVA was conducted for the two experiments involving constant and alternating temperatures. The factors included temperature, light, and storage times. After ANOVA, means were separated using the least significant difference (LSD) method at probability level of P ≤ 0.05..

In the light/dark treatment, daily seed counts were recorded, and the relationship between imbibition time and cumulative germination percentage was assessed using regression analysis. For the continuous darkness treatment, where daily counting was omitted to avoid potential errors from accidental light exposure, only the final germination count was taken, followed by an analysis of variance (ANOVA).

The relationship between imbibition time and cumulative germination at different temperatures and storage times under light/dark regime was fitted using a three-parameter sigmoid model in Sigma Plot 14.0. The model (Eq 1) is defined as follows.


$$G=G_{max}/[(\text{1}+e-(T-T_{\text{5}\text{0}})]/G_{rate}])$$ (1)

Where G is the cumulative of germination (%) at times T, G_max_ is the maximum germination (%), T_50_ is the times (day) required to reach 50% of the maximum germination, and G_rate_ indicates the slope.

Germination percentages from both NaCl and osmotic potential experiments were fitted to a three-parameter logistic model using SigmaPlot software (version 14).


$$G=G_{max}/[\text{1}+(x/x_{\text{5}\text{0}}{)}^{\wedge }Grate]\;$$ (2)

where, G is the total germination (%) at NaCl concentration or osmotic potential x, G_max_ is the maximum germination (%), X_50_ is the NaCl concentration or osmotic potential for 50% inhibition of the maximum germination (%), and G_rate_ is the slope.

## References

[1] Sahrawat A, Sharma J, Jawla SK. Evaluation of Antioxidant and Antifungal Activity of the Whole Part of Dactyloctenium aegyptium Weed on Bengal Gram. LR. 2021;(Of). doi: 10.18805/lr-4749

[2] Chauhan BS. Crowfootgrass (Dactyloctenium aegyptium) Germination and Response to Herbicides in the Philippines. Weed sci. 2011;59(4):512–6. doi: 10.1614/ws-d-11-00048.1

[3] Holm LG, Plucknett DL, Pancho JV, Herberger JP. The world’s worst weeds. Distribution and biology. University Press of Hawaii. 1977.

[4] Galinato MI. Upland rice weeds of South and Southeast Asia. Int. Rice Res. Inst. 1999.

[5] Quintana M, Chantre GR, Reinoso O, Renzi JP. Population Parameters as Key Factors for Site-Specific Distribution of Invasive Weed Rhynchosia senna in Semiarid Temperate Agroecosystems. Agronomy. 2025;15(4):858. doi: 10.3390/agronomy15040858

[6] Pham DH, Wu CA. Seed longevity and germination of the emerging invasive species wavyleaf basketgrass (Oplismenus undulatifolius) under varied light regimes. Invasive plant sci manag. 2023;16(4):225–32. doi: 10.1017/inp.2023.27

[7] Javaid MM, Mahmood A, Alshaya DS, AlKahtani MDF, Waheed H, Wasaya A, et al. Influence of environmental factors on seed germination and seedling characteristics of perennial ryegrass (Lolium perenne L.). Sci Rep. 2022;12(1):9522. doi: 10.1038/s41598-022-13416-6 35681016 PMC9184647

[8] Chen Y, Masoom A, Huang Z, Xue J, Chen G. Interspecific and intraspecific differences in seed germination response to different temperatures of three Echinochloa rice weeds: a case study with 327 populations. Weed Science. 2025;73:e23. doi: 10.1017/wsc.2024.104

[9] Presotto A, Poverene M, Cantamutto M. Seed dormancy and hybridization effect of the invasive species, Helianthus annuus. Annals of Applied Biology. 2014;164(3):373–83. doi: 10.1111/aab.12104

[10] Vandelook F, Van de Moer D, Van Assche JA. Environmental signals for seed germination reflect habitat adaptations in four temperate Caryophyllaceae. Functional Ecology. 2008;22(3):470–8. doi: 10.1111/j.1365-2435.2008.01385.x

[11] Bentsink L, Koornneef M. Seed dormancy and germination. Arabidopsis Book. 2008;6:e0119. doi: 10.1199/tab.0119 22303244 PMC3243337

[12] Li J, Li G, Wang H, Wang Deng X. Phytochrome signaling mechanisms. Arabidopsis Book. 2011;9:e0148. doi: 10.1199/tab.0148 22303272 PMC3268501

[13] Ahmed S, Opeña JL, Chauhan BS. Seed Germination Ecology of Doveweed (Murdannia nudiflora)and Its Implication for Management in Dry-Seeded Rice. Weed sci. 2015;63(2):491–501. doi: 10.1614/ws-d-14-00115.1

[14] Ntanasi T, Karavidas I, Zioviris G, Ziogas I, Karaolani M, Fortis D, et al. Assessment of Growth, Yield, and Nutrient Uptake of Mediterranean Tomato Landraces in Response to Salinity Stress. Plants (Basel). 2023;12(20):3551. doi: 10.3390/plants12203551 37896015 PMC10610299

[15] Rojas‐Aréchiga M, García‐Morales E. Dormancy and viability of Ferocactus peninsulae (Cactaceae) seeds. Plant Species Biology. 2022;37(2):173–81. doi: 10.1111/1442-1984.12365

[16] Née G, Xiang Y, Soppe WJ. The release of dormancy, a wake-up call for seeds to germinate. Current Opinion in Plant Biology. 2017;35:8–14. doi: 10.1016/j.pbi.2016.09.002 27710774

[17] Humphries T, Chauhan BS, Florentine SK. Environmental factors effecting the germination and seedling emergence of two populations of an aggressive agricultural weed; Nassella trichotoma. PLoS One. 2018;13(7):e0199491. doi: 10.1371/journal.pone.0199491 29975730 PMC6033418

[18] Cochavi A, Goldwasser Y, Horesh A, Igbariya K, Lati RN. Impact of environmental factors on seed germination and emergence of wild poinsettia (Euphorbia geniculata Ortega). Crop Protection. 2018;114:68–75. doi: 10.1016/j.cropro.2018.08.019

[19] Chauhan BS, Johnson DE. The Role of Seed Ecology in Improving Weed Management Strategies in the Tropics. Advances in Agronomy. Elsevier. 2010. 221–62. doi: 10.1016/s0065-2113(10)05006-6

[20] Mausbach J, Irmak S, Chahal P, Sarangi D, Jhala AJ. Effect of degree of water stress on growth and fecundity of velvetleaf (Abutilon theophrasti) using soil moisture sensors. Weed Sci. 2022;70(6):698–705. doi: 10.1017/wsc.2022.54

[21] Zhang R, Baskin JM, Baskin CC, Mo Q, Chen L, Hu X, et al. Effect of population, collection year, after-ripening and incubation condition on seed germination of Stipa bungeana. Sci Rep. 2017;7(1):13893. doi: 10.1038/s41598-017-14267-2 29066779 PMC5655671

[22] Qaderi MM. Environmental Regulation of Weed Seed Dormancy and Germination. Seeds. 2023;2(3):259–77. doi: 10.3390/seeds2030020

[23] Andersson L, Milberg P. Variation in seed dormancy among mother plants, populations and years of seed collection. Seed Sci Res. 1998;8(1):29–38. doi: 10.1017/s0960258500003883

[24] Singh M, Thapa R, Kukal MS, Irmak S, Mirsky S, Jhala AJ. Effect of water stress on weed germination, growth characteristics, and seed production: a global meta-analysis. Weed Sci. 2022;70(6):621–40. doi: 10.1017/wsc.2022.59

[25] Burke IC, Thomas WE, Spears JF, Wilcut JW. Influence of environmental factors on after-ripened crowfootgrass (Dactyloctenium aegyptium) seed germination. Weed Science. 2003;51(3):342–7. doi: 10.1614/0043-1745(2003)051[0342:ioefoa]2.0.co;2

[26] Okusanya OT, Sonaike AA. Germination behaviour of Dactyloctenium aegyptium from two localities in Nigeria. Physiol Plant. 1991;81(4):489–94. doi: 10.1034/j.1399-3054.1991.810407.x

[27] Adu AA, Yeo AR, Okusanya OT. The response to salinity of a population of Dactyloctenium aegyptium from a saline habitat in southern Nigeria. J Trop Ecol. 1994;10(2):219–28. doi: 10.1017/s0266467400007860

[28] Baskin CC, Baskin JM. Breaking Seed Dormancy during Dry Storage: A Useful Tool or Major Problem for Successful Restoration via Direct Seeding?. Plants (Basel). 2020;9(5):636. doi: 10.3390/plants9050636 32429336 PMC7284515

[29] Soltani E, Baskin CC, Gonzalez-Andujar JL. An Overview of Environmental Cues That Affect Germination of Nondormant Seeds. Seeds. 2022;1(2):146–51. doi: 10.3390/seeds1020013

[30] Chiu RS, Nahal H, Provart NJ, Gazzarrini S. The role of the Arabidopsis FUSCA3 transcription factor during inhibition of seed germination at high temperature. BMC Plant Biol. 2012;12:15. doi: 10.1186/1471-2229-12-15 22279962 PMC3296646

[31] Ali-Rachedi S, Bouinot D, Wagner M-H, Bonnet M, Sotta B, Grappin P, et al. Changes in endogenous abscisic acid levels during dormancy release and maintenance of mature seeds: studies with the Cape Verde Islands ecotype, the dormant model of Arabidopsis thaliana. Planta. 2004;219(3):479–88. doi: 10.1007/s00425-004-1251-4 15060827

[32] Schutz W, Milberg P, Lamont BB. Seed dormancy, after-ripening and light requirements of four annual Asteraceae in south-western Australia. Ann Bot. 2002;90(6):707–14. doi: 10.1093/aob/mcf250 12451026 PMC4240361

[33] Benvenuti S. Soil Texture Involvement in Germination and Emergence of Buried Weed Seeds. Agronomy Journal. 2003;95(1):191. doi: 10.2134/agronj2003.0191

[34] Tang W, Guo H, Yin J, Ding X, Xu X, Wang T, et al. Germination ecology of Chenopodium album L. and implications for weed management. PLoS One. 2022;17(10):e0276176. doi: 10.1371/journal.pone.0276176 36251670 PMC9576060

[35] Manalil S, Haider Ali H, Chauhan BS. Germination ecology of turnip weed (Rapistrum rugosum (L.) All.) in the northern regions of Australia. PLoS One. 2018;13(7):e0201023. doi: 10.1371/journal.pone.0201023 30024963 PMC6053197

[36] Du C, Li L, Effah Z. Effects of Straw Mulching and Reduced Tillage on Crop Production and Environment: A Review. Water. 2022;14(16):2471. doi: 10.3390/w14162471

[37] Minhas WA, Mehboob N, Yahya M, Rehman HU, Farooq S, Hussain M. The Influence of Different Crop Mulches on Weed Infestation, Soil Properties and Productivity of Wheat under Conventional and Conservation Production Systems. Plants (Basel). 2022;12(1):9. doi: 10.3390/plants12010009 36616137 PMC9824819

[38] Scavo A, Mauromicale G. Crop Allelopathy for Sustainable Weed Management in Agroecosystems: Knowing the Present with a View to the Future. Agronomy. 2021;11(11):2104. doi: 10.3390/agronomy11112104

[39] Kanatas P, Gazoulis I, Petraki D, Kokkini M, Antonopoulos N, Travlos I. False Seedbed and Stale Seedbed Against Important Broadleaf Weeds: A Case Study and a Step Closer to Agroecology. Plants (Basel). 2025;14(4):564. doi: 10.3390/plants14040564 40006823 PMC11859391

[40] Singh M, Bhullar MS, Gill G. Integrated weed management in dry-seeded rice using stale seedbeds and post sowing herbicides. Field Crops Research. 2018;224:182–91. doi: 10.1016/j.fcr.2018.03.002

[41] Chauhan BS, Johnson DE. Seed Germination Ecology of Junglerice (Echinochloa colona): A Major Weed of Rice. Weed sci. 2009;57(3):235–40. doi: 10.1614/ws-08-141.1

[42] Chauhan BS, Johnson DE. Germination, emergence, and dormancy of Mimosa pudica. Weed Biology and Management. 2009;9(1):38–45. doi: 10.1111/j.1445-6664.2008.00316.x

[43] Chauhan BS. Germination biology of liverseedgrass (Urochloa panicoides) and its response to postemergence herbicides in Australian conditions. Weed Sci. 2022;70(5):553–60. doi: 10.1017/wsc.2022.50

[44] Han Y, Gao H, Wang Y, Zhang L, Jia J, Ma H. Storage Time Affects the Viability, Longevity, and Germination of Eriochloa villosa (Thunb.) Kunth Seeds. Sustainability. 2023;15(11):8576. doi: 10.3390/su15118576

[45] Zhou YM, Lu JJ, Tan DY, Baskin CC, Baskin JM. Seed Germination Ecology of the Cold Desert Annual Isatis violascens (Brassicaceae): Two Levels of Physiological Dormancy and Role of the Pericarp. PLoS One. 2015;10(10):e0140983. doi: 10.1371/journal.pone.0140983 26513241 PMC4626048

[46] Cristaudo A, Gresta F, Luciani F, Restuccia A. Effects of after‐harvest period and environmental factors on seed dormancy of Amaranthus species. Weed Research. 2007;47(4):327–34. doi: 10.1111/j.1365-3180.2007.00574.x

[47] Mwando E, Han Y, Angessa TT, Zhou G, Hill CB, Zhang X-Q. Genome-wide association study of salinity tolerance during germination in barley (Hordeum vulgare L.). Frontiers in Plant Science. 2020;11:118. doi: 10.3389/fpls.2020.00118 32153619 PMC7047234

[48] Ibrahim EA. Seed priming to alleviate salinity stress in germinating seeds. J Plant Physiol. 2016;192:38–46. doi: 10.1016/j.jplph.2015.12.011 26812088

[49] Mobli A, Mollaee M, Manalil S, Chauhan BS. Germination Ecology of Brachiaria eruciformis in Australia and Its Implications for Weed Management. Agronomy. 2019;10(1):30. doi: 10.3390/agronomy10010030

[50] Li Q, Tan J, Li W, Yuan G, Du L, Ma S, et al. Effects of Environmental Factors on Seed Germination and Emergence of Japanese Brome (Bromus japonicus). Weed sci. 2015;63(3):641–6. doi: 10.1614/ws-d-14-00131.1

[51] Singh M, Ramirez AHM, Sharma SD, Jhala AJ. Factors Affecting the Germination of Tall Morningglory (Ipomoea purpurea). Weed sci. 2012;60(1):64–8. doi: 10.1614/ws-d-11-00106.1

[52] Javaid MM, Mahmood A, Alshaya DS, AlKahtani MDF, Waheed H, Wasaya A, et al. Influence of environmental factors on seed germination and seedling characteristics of perennial ryegrass (Lolium perenne L.). Sci Rep. 2022;12(1):9522. doi: 10.1038/s41598-022-13416-6 35681016 PMC9184647

[53] Toscano S, Romano D, Tribulato A, Patanè C. Effects of drought stress on seed germination of ornamental sunflowers. Acta Physiol Plant. 2017;39(8). doi: 10.1007/s11738-017-2484-8

[54] Ferrari FN, Parera CA. Germination of six native perennial grasses that can be used as potential soil cover crops in drip-irrigated vineyards in semiarid environs of Argentina. Journal of Arid Environments. 2015;113:1–5. doi: 10.1016/j.jaridenv.2014.09.002

[55] Michel BE. Evaluation of the water potentials of solutions of polyethylene glycol 8000 both in the absence and presence of other solutes. Plant Physiol. 1983;72(1):66–70. doi: 10.1104/pp.72.1.66 16662983 PMC1066170

