# Supplementary material for: Adaptive strategies of Egyptian crowfoot grass (Dactyloctenium aegyptium (L.) Willd): Germination responses to environmental factors
Source: PLoS One. 2026 May 7;21(5):e0345587. doi: 10.1371/journal.pone.0345587 (PMC13152129; doi:10.1371/journal.pone.0345587)
Supplement: Attachment — Submitted filename: Response to reviewers.docx [file pone.0345587.s003.docx]

Response to reviewers.

Paper ID: PONE-D-25-58145

**Adaptive Strategies of Egyptian Crowfoot Grass (*Dactyloctenium aegyptium* (L.) Willd): Germination Responses to Environmental Factors**

Dear Dr. xxxxxxxxxxxxxxxxxx,

We wish to thank you and the unknown reviewers for reviewing of our manuscript (PONE-D-25-58145) and the valuable comments which helped us to improve the quality of our paper. The manuscript was revised based on the comments. In the following, we have provided a point-by-point reply to the questions and comments, where the questions/comments and answers were written in “yellow” and “green”, respectively. In addition to the cleaned version of the manuscript, we have included a version with highlight yellow for Reviewer.

Question: Have any other tests been performed outside of the laboratory? If not, no laboratory statement is required.

Answer: Thank you for your comment. We confirm that no tests were performed outside the laboratory setting. All experimental procedures and analyses were conducted under controlled laboratory conditions at Agricultural Sciences and Natural Resources University of Khuzestan.

Question: My suggestion for the sentence: the effects of constant and alternating temperatures, storage times, light, salinity, and water stress on germination of Egyptian crowfoot grass studied under laboratory conditions. laboratory statement is required.

Answer: In response to the reviewer’s helpful suggestion, the sentence has been revised accordingly

Question: I recommend checking the rules of reference.

Answer: The reference has been corrected and incorporated into the text

Question: Is it '','' or ''-'' here?

The references in this manuscript were prepared using EndNote and formatted in accordance with the Vancouver style. Specifically, when citing three or more consecutive references in the text, we have used an en dash (–) to indicate the range (e.g., [5–7]), as per standard Vancouver conventions

Question:I don't think you need to specify this statement separately in parentheses.

Answer :The abbreviation in the manuscript text has been removed.

Question: start with three ways of interaction. there was no interaction among three tretments as well as light*storage on germination of ..... The effct of temperture was changed by light or storage time. You can mention more properly.

Answer :We appreciate your helpful suggestion, and the manuscript has been revised accordingly

Question: You can provide a brief explanation, in the form of a comment, as to why these factors are not effective. This would be helpful for clarity.

Answer :Revised per the reviewer’s recommendation

Question: highest seems better wording

Answer :Revised as suggested by the reviewer to use 'highest' for improved clarity

Question: instead it can be written "longer storage times". No need to mention same words again and again. This apply for other words also.

Answer :Revised

Question: The ineffectiveness of light on storage was mentioned above, but germination decreased in the absence of light. Wasn't the decrease statistically significant?

Answer :The following explanatory text has been incorporated into the manuscript

Question: Do you think the result would have changed if you tried 40 ºC?

Answer :The 40 °C treatment was included in the experimental design; however, germination was completely inhibited at this temperature (0% germination), indicating that thermal conditions at or above 40 °C are non-permissive for germination in this species.

Question: shorter can be better word.

Answer :Revised

Question: The subheadings are too long, I think it would be better if you wrote them briefly.

Answer : Revised

Question: please see my comment on former ANOVA

Answer : Revised and rephrased in response to the reviewer’s comments.

Question: I assume you set the temperature for day and night. But you always get a 10°C difference. Did you adjust the temperature values based on the areas where this weed is located?

Answer: Thank you for your insightful and precise question. In the majority of published studies examining alternating temperature regimes—particularly in seed germination research a 10 °C day/night thermal differential is widely employed as a standard experimental protocol. In our study, this amplitude was deliberately selected not only for consistency with established methodologies but also to approximate realistic field conditions in the region where Egyptian crowfoot grass is prevalent. This species typically initiates germination and establishment in late spring a period when post-irrigation soil temperatures exhibit diurnal fluctuations that closely correspond to the temperature ranges applied in Experiment alternating temperature.

Question: Since temperature values vary by region, it would be better if you write the average temperature values for that period in parentheses next to the month.

Answer :Mean temperatures have been incorporated into the manuscript in accordance with the reviewer’s suggestion

Question:The transition from the previous paragraph to this one is quite sharp. Let's start the paragraph by adding a sentence of explanation.

Answer: Revised and rephrased in response to the reviewer’s comments.

Question: This is the first time this type of name has been used, the author's name should also be added.

Answer: Revised

Question: Check the species names throughout the article. Add the author's name to those used for the first time.

Answer: Revised

Question: Check all references that contain '','' or ''-''.

Question:If such names are used for the first time, they cannot be abbreviated.

Answer: Revised

Question:If such names are used for the first time, they cannot be abbreviated.

Answer: Revised

Question :There are differences in the spacing between paragraphs. Apply the same rule to all paragraphs.

Answer:Thank you for highlighting this issue. Paragraph spacing has been uniformly adjusted throughout the manuscript to ensure consistent formatting in line with journal guidelines.

Question :highly can be better word

Revised

Question How was it collected? How was it cleaned and made ready for use? How was it stored?

Answer: Additional details have been incorporated. Also The storage conditions, moisture content, and other relevant details have already been described in the text

Question: Was dormancy broken during this time? If so, please mention the germination in the control group.

Answer:No dormancy-breaking treatment was applied

Question: As I mentioned before, subheadings should be shortened in this section.

Revised

Question: what is the difference between the first sentences (2 lines up) and this

deleted

Question: Even if these temperature values give a result of zero, it would be better to see it in the results section.

Answer:In response to the reviewer’s comment, data for 5, 10, and 40 °C were incorporated into the analysis. The dataset was re-analyzed by ANOVA, followed by post-hoc mean comparisons, and figures were updated accordingly."

Question: Coincidence plots?

Answer: The authors appreciate the reviewer’s comment. If the question refers to the experimental design, we clarify that the study was conducted under controlled conditions using a factorial arrangement within a completely randomized design (CRD)."

Question: ‘’ <’’ or ‘’=’’ ?

Answer:Revised

Question:If you align the formulas, they will be easier to read at first glance.

Answer:Revised

Question:The "c.v." value is a bit high. Can you explain why you think so?

Answer:We agree with the reviewer that, under standard laboratory conditions, the coefficient of variation (CV) typically ranges from 10 to 15%. However, the inclusion of extreme temperatures (5, 10, and 40 °C) and the high variability in germination under darkness—especially at 35 °C—resulted in an elevated CV. This was driven by inconsistent germination among replicate Petri dishes, where some showed no germination while others exhibited substantial germination."

Question:Also pay attention to italics in references.

Answer: References were managed using EndNote; minor formatting inconsistencies may have arisen due to repeated editing and reopening of the Word document. Since the article’s references may be altered again when EndNote is updated, the full reference list has been copied verbatim at the end of the article.

The authors sincerely thank the reviewers for their thoughtful evaluation and insightful comments, which have greatly enhanced the quality of this manuscript. Any additional suggestions or requested revisions will be implemented promptly

Ahmad Zare
